# 3D Analysis of HCMV Induced-Nuclear Membrane Structures by FIB/SEM Tomography: Insight into an Unprecedented Membrane Morphology

**DOI:** 10.3390/v7112900

**Published:** 2015-11-04

**Authors:** Clarissa Villinger, Gregor Neusser, Christine Kranz, Paul Walther, Thomas Mertens

**Affiliations:** 1 Electron Microscopy Facility, Ulm University, Albert-Einstein-Allee 11, 89081 Ulm, Germany; clarissa.villinger@uni-ulm.de; 2 Institute of Virology, University Medical Center Ulm, Albert-Einstein-Allee 11, 89081 Ulm, Germany; thomas.mertens@uniklinik-ulm.de; 3 Institute of Analytical and Bioanalytical Chemistry, Ulm University, Albert-Einstein-Allee 11, 89081 Ulm, Germany; gregor.neusser@uni-ulm.de (G.N.); christine.kranz@uni-ulm.de (C.K.)

**Keywords:** FIB/SEM tomography, HCMV, nuclear capsid egress, inner nuclear membrane infoldings, high-pressure freezing, three-dimensional structure

## Abstract

We show that focused ion beam/scanning electron microscopy (FIB/SEM) tomography is an excellent method to analyze the three-dimensional structure of a fibroblast nucleus infected with human cytomegalovirus (HCMV). We found that the previously described infoldings of the inner nuclear membrane, which are unique among its kind, form an extremely complex network of membrane structures not predictable by previous two-dimensional studies. In all cases they contained further invaginations (2nd and 3rd order infoldings). Quantification revealed 5498 HCMV capsids within two nuclear segments, allowing an estimate of 15,000 to 30,000 capsids in the entire nucleus five days post infection. Only 0.8% proved to be enveloped capsids which were exclusively detected in 1st order infoldings (perinuclear space). Distribution of the capsids between 1st, 2nd and 3rd order infoldings is in complete agreement with the envelopment/de-envelopment model for egress of HCMV capsids from the nucleus and we confirm that capsid budding does occur at the large infoldings. Based on our results we propose the *pushing membrane model*: HCMV infection induces local disruption of the nuclear lamina and synthesis of new membrane material which is pushed into the nucleoplasm, forming complex membrane infoldings in a highly abundant manner, which then may be also used by nucleocapsids for budding.

## 1. Introduction

Human cytomegalovirus (HCMV) morphogenesis has been studied extensively by transmission electron microscopy (TEM) [1,2,3,4,5,6,7]. The large HCMV virion can be identified unambiguously in micrographs and the life cycle of HCMV proceeds mainly within defined and characteristic compartments of the host cell, namely within the nucleus and in a cytoplasmatic site referred to as the “viral assembly complex”. As a member of the *betaherpesvirinae* subfamily, HCMV particles consist of (I) the capsid, a stable protein shell that harbors the viral genome; (II) the tegument, a proteinaceous layer associated with the capsid; and (III) the virus envelope, a lipid bilayer originating from the host cell, which surrounds the tegumented capsid. More than 70 virally encoded proteins and cellular proteins contribute to the virus structure [8,9,10]. This study focuses on the nuclear stage of replication. The enlarged host cell nucleus is the site of capsid formation as well as DNA synthesis and encapsidation. The spherical, unstable procapsid assembles around scaffold proteins. Spontaneous angularization then leads to stable icosahedral capsids which are ready for DNA encapsidation. There are three different capsid maturation forms: B capsids contain the scaffold that appears as a ring in TEM images. During DNA encapsidation the scaffold is degraded, resulting in DNA-filled C capsids. In some cases the scaffold is degraded and DNA encapsidation does not take place which leads to the formation of A capsids [11,12,13,14]. Whether A and B capsids are abortive capsid forms or intermediates during formation of C capsids has not been clearly answered so far [12]. The nuclear stage ends with the transition of tegumented capsids from the nucleus into the cytoplasm where final virus tegumentation and envelopment occurs in the viral assembly complex.

Virus capsids with a diameter of ~85 nm cannot be transported through intact nuclear pores [15]. The nuclear envelope, consisting of the inner and outer nuclear membrane and a layer of nuclear lamins, forms an obstacle on the way into the cytoplasm. The debate on the mechanism of nuclear egress of different herpesviruses is still controversial [16,17,18,19,20], but the model of nuclear egress by primary envelopment at the inner nuclear membrane and de-envelopment at the outer nuclear membrane leading to non-enveloped capsids in the cytoplasm [17] has been widely accepted. The essential [21,22] HCMV proteins pUL50 and pUL53 recruit kinases for local degradation of the nuclear lamina by phosphorylation of lamins [23,24,25]. Transport across the nuclear envelope by envelopment and de-envelopment of the “cargo” has also been described for the transport of ribonucleoprotein complexes in drosophila synapses ([26,27], reviewed in [19]). This could indicate that HCMV hijacks an already existing cellular transport mechanism.

We previously presented evidence that the HCMV strain AD169 and the MCMV Smith strain induce large infoldings of the inner nuclear membrane in fibroblasts [1]. Such large infoldings were also observed in macrophages infected with the HCMV strain TB40E [28]. Recently Malhas *et al.* [29] suggested a nomenclature for nuclear membrane structures, termed nucleoplasmic reticulum (NR). By their definition NR type I is “an invagination of the inner nuclear membrane alone” which “do(es) not contain a cytoplasmic core” and is “only incompletely ensheathed by a nuclear lamina. They can be branched and in extreme examples can be stacked and extensively ramified within the nucleoplasm”. In contrast to that, Type II NR is defined as “a double-membrane-walled invagination of the inner and outer nuclear membranes enclosing a diffusion-accessible cytoplasmic core. (…) The cytoplasmic core often contains cytoskeletal elements, including microfilaments, intermediate filament and microtubules as well as ribosomes and small vesicles” [29]. The infoldings described by Buser *et al.* [1] correspond to NR type I. Buser *et al.* [1] suggested that these infoldings, which seemed to be free of nuclear lamina, provide a route for capsid egress without obstacles such as chromatin and nucleoli.

The major limitation of our previous study was that the three-dimensional model of the infolding structure was based on two-dimensional TEM micrographs and therefore remained hypothetical [1]. Several electron microscopic methods for three-dimensional visualization are available to date: (I) tilt series based electron tomography; (II) serial sectioning TEM; (III) serial block face scanning electron microscopy (SBF-SEM); and (IV) focused ion beam/scanning electron microscopy (FIB/SEM) tomography. For the last decade FIB/SEM tomography has increasingly contributed to three-dimensional visualization of biological specimens [30,31,32,33,34,35,36,37]. For the first time we used FIB/SEM tomography to visualize a large part of an HCMV infected nucleus (dimensions of several µm) with a resolution in the z direction of 20 nm. We recently published the proof of principle study that FIB/SEM tomography of adherent cells generates high-resolution datasets with good image contrast [38,39].

By visualizing the three-dimensional structure of an HCMV-infected nucleus and the nuclear infoldings with FIB/SEM tomography, we could quantitatively determine the high number of nuclear capsids and their association with the different infoldings, providing new evidence for the envelopment/de-envelopment mechanism for nuclear egress of HCMV capsids. Furthermore, we could identify a network of nuclear membrane structures that not only corresponds to the definition of NR type I but also expands the definition by its hierarchical architecture and interlaced nature. Finally, we propose a mechanism that could explain the formation of such complex nuclear membrane structures.

## 2. Materials and Methods

### 2.1. Cells and Virus

Human foreskin fibroblasts (HFFs) were maintained in minimal essential medium (Gibco, ThermoFisher Scientific Inc., Waltham, MA, USA) supplemented with 10% fetal calf serum (Gibco), 2 mM l-glutamine (200 mM; PAA Laboratories GmbH, Pasching/Linz, Austria), 1% penicillin-streptomycin (100-fold; PAA Laboratories) and 1% non-essential amino acids (Biochrom AG, Berlin, Germany) and were used before passage 23. For FIB/SEM and TEM experiments HFFs were seeded on carbon coated and glow discharged sapphire discs (50 µm thick, Engineering Office M. Wohlwend GmbH, Sennwald, Switzerland). After cell attachment for 24 h they were infected with the HCMV bacterial artificial chromosome (BAC) clone TB40-BAC4 [40] with a multiplicity of infection (MOI) of 1 and incubated for three or five days (37 °C, 5% CO_2_).

### 2.2. Sample Preparation for FIB/SEM Tomography and Transmission Electron Microscopy (TEM)

EM sample preparation was conducted by high-pressure freezing, freeze substitution and Epon embedding as described earlier [1,41]. For that the sapphire disc (Engineering Office M. Wohlwend GmbH) with the infected cells was clamped between two aluminum planchettes, so that the cells were protected in the 100 µm cavity of the upper planchette. The sandwich was then high-pressure frozen (HPF Compact 01, Engineering Office M. Wohlwend GmbH), using hexadecene as a filler for improved conductivity of pressure and temperature. Freeze substitution and Epon embedding were performed with a substitution medium consisting of acetone with 0.2% osmium tetroxide (*wt*/*vol*), 0.1% uranyl acetate (*wt*/*vol*) and 5% (*vol*/*vol*) of water for good membrane contrast [42]. The temperature was exponentially raised from −90 °C to 0 °C within 17 h. The samples were then brought to room temperature and washed twice with acetone followed by stepwise embedding in Epon. After polymerization at 60 °C within 72 h the sapphire disc could then be removed from the embedded sample, so that the cells remained directly underneath the surface of the Epon block.

### 2.3. FIB/SEM Tomography

**Figure 1:**
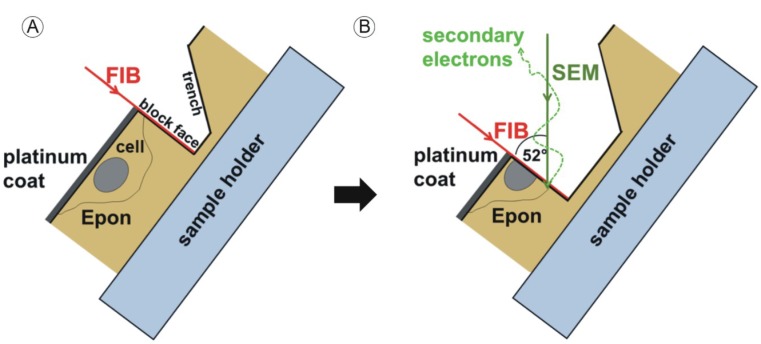
Sample manipulation by the focused ion beam (FIB) and image acquisition with the scanning electron microscope (SEM) beam. Sample preparation is described in [39]. Embedded HCMV infected fibroblasts are located directly under the surface of the approximately 1 mm high Epon block. The sample is coated with a thin platinum layer prior to mounting into the dual beam microscope. The area of interest is chosen at 10 kV acceleration voltage. (**A**) The sample is then tilted to 52° and a trench is FIB-milled into the Epon block, generating a new surface (block face); (**B**) Removal of the next thin layer (“slice”) of the sample generates a new block face. After every “slice” step the generated block face is imaged by the scanning electron beam (“view”). The “slice and view” cycle is repeated to gain a three-dimensional dataset of the volume of interest. Reprinted from [38] Figure 1 with kind permission from Springer Science+Business Media.

First, we sawed the Epon block into two halves. One half was objected to serial ultrathin sectioning (see 2.5.). As described in [38,39], we reduced the size of the second half with a jigsaw to a height of ~1 mm. The produced Epon disc was then mounted onto a SEM specimen stub (Figure 1). The sample was then coated with 5 nm of platinum using an electron beam evaporator (Baltec, Balzers, Liechtenstein) to enhance electrical conductivity. FIB/SEM tomography was conducted with a Helios Nanolab 600 FIB/SEM (FEI, Eindhoven, The Netherlands). The sample surface was imaged at a high acceleration voltage of 10 kV to visualize the contours of the embedded cells. In order to protect the cellular structures in the chosen area from beam damage during FIB-milling, the surface was covered with an additional platinum layer using ion beam-induced deposition based on the fragmentation of methylcyclopentadienyl(trimethyl)platinum (C_9_H_16_Pt).

Platinum deposition was first conducted at 30 kV and a low current for a few minutes until a first thin protecting layer was formed. Subsequently, a thicker (~300 nm) coating was deposited at a higher beam current. A block face was then generated to gain access to the internal structures of the chosen cell by milling a regular cross section (perpendicular to the cell substrate and resin surface). After a cleaning cross section the area for the slice and view process was defined. Slice and view was performed using the software module Auto Slice & View.G1 (FEI). Slicing was obtained at 30 kV with a beam current of 93 pA using the provided material file for PMMA. With each step, 20 nm of the material was removed by the FIB and the newly produced block face was imaged with the SEM at an accelerating voltage of 5 kV using the secondary electron signal recorded with the through-the-lens detector. The pixel dimensions of a recorded image were set to 2048 × 1768 pixel (6.4 µm × 5.5 µm). That equals a pixel size of approximately 3 nm. Recording of a single image took 36.2 s.

### 2.4. Data Processing

First, the images were rotated 180° so that the cell substrate was located at the bottom of the image and the contrast was inversed. Contrast and brightness adjustment was performed with the open source software ImageJ [43]. The open source software IMOD [44,45] was used for automatic alignment of the images. Manual improvement of the alignment and segmentation was then conducted using Avizo 6.3 (FEI Visualization Science Group, Burlington, MA, USA). The nuclear membrane and the capsids were segmented manually. The space between two images (20 nm) was considerably smaller than the diameter of a capsid (about 85 nm). Therefore, one capsid was visible in more than one image. For segmentation, every capsid was only labelled on one image. Quantification of the different capsid types in the three-dimensional reconstruction was done automatically in Avizo.

### 2.5. (Serial-)Ultrathin Sectioning and TEM

TEM was performed to test the quality of the sample prior to FIB/SEM tomography, to compare the resolution of TEM and FIB/SEM images and to collect (serial-)TEM images of nuclear infoldings of virus infected cells three and five days post infection. For that, (serial-)ultrathin sections (thickness of 80 nm, for serial sectioning 90 nm) were produced from the respective Epon block with an ultramicrotome (Ultracut UCT, Leica) equipped with a diamond knife (Diatome, Biel, Switzerland). The second half of the Epon block that was examined by FIB/SEM tomography was used for serial sectioning (described in [41]). The sections were imaged with a JEOL-1400 TEM (JEOL,  Akishima, Tokyo, Japan) at 120 kV.

## 3. Results

### 3.1. FIB/SEM Tomography is a Powerful Tool for Virological Research

We were able to visualize the detailed structure of a defined large volume of a representative HCMV-infected nucleus in three dimensions using FIB/SEM tomography to clarify the morphology of the infoldings of the inner nuclear membrane within the nucleus to elucidate the process of infolding formation. The focused ion beam (FIB) removes specimen material with an accuracy of a few nanometers (“slice”) and makes the underlying structures accessible for the scanning electron beam (Figure 1) that images the newly produced surface (“view”), the “block face”. The cycle of “slice and view” is repeated until the volume of interest is completely imaged section by section. One major advantage of the method is the high resolution in the z direction which allows the complete quantitative determination of all virus capsids within the analyzed segment of the nucleus.

**Figure 2:**
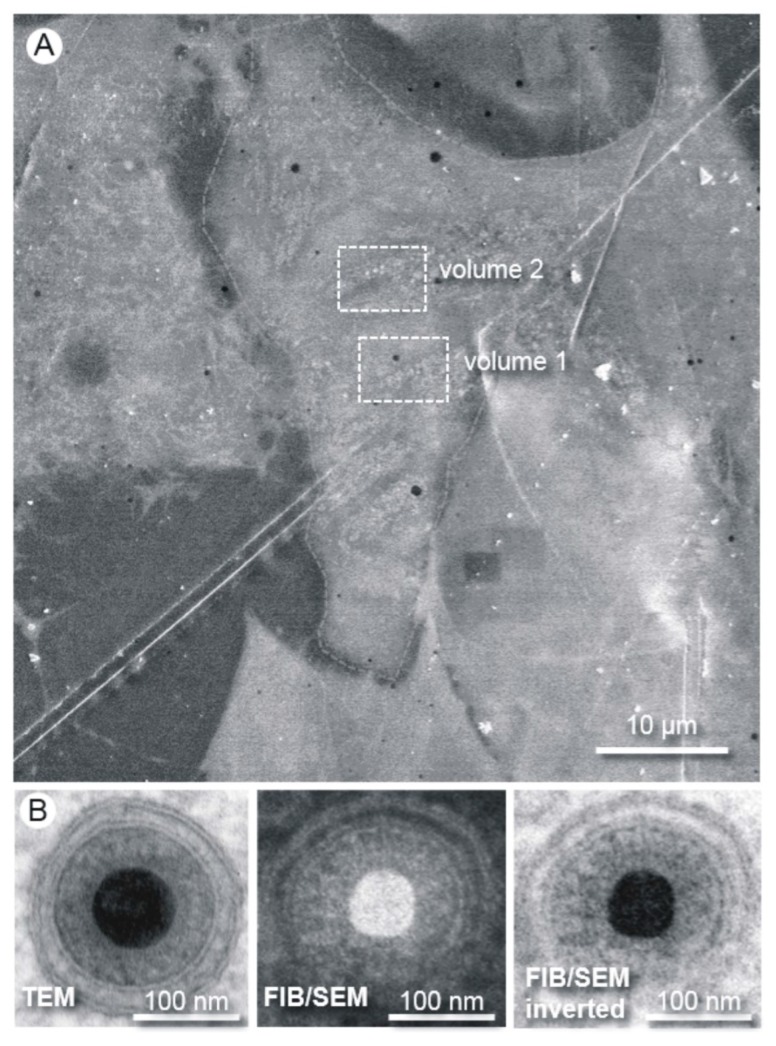
Methodology and resolution of FIB/SEM tomography. (**A**) The Epon embedded cells are first visualized by SEM at an acceleration voltage of 10 kV. A region of interest can be easily chosen for the subsequent “slice and view” process. The boxes show the positions of the volumes chosen for FIB/SEM tomography; (**B**) Images of human cytomegalovirus (HCMV) particles acquired from an ultrathin section by transmission electron microscopy (TEM) and from the block face by FIB/SEM. The high resolution of the FIB/SEM image allows clear visibility of the two leaflets of the lipid bilayer. The secondary electron SEM image was acquired with an acceleration voltage of 5 kV.

We analyzed two segments of one infected fibroblast nucleus at five days post infection (Figure 2A). FIB/SEM proved to be ideal for gaining high quality structural information of adherent cells and allowed the selection of an optimum region before starting the “slice and view” process (Figure 2A). Both analyzed volumes had the same dimensions (6.4 µm × 5.5 µm × 5 µm) and each dataset consisted of 250 subsequent images from the slice and view processes with an increment of 20 nm. The three-dimensional reconstruction was based on stacking the obtained images to a tomogram. With our advanced specimen preparation and FIB/SEM imaging approach using the secondary electron signal at 5 kV acceleration voltage, we gained a resolution comparable to standard TEM (Figure 2B).

### 3.2. Three Dimensional FIB/SEM Tomography Reveals the Enormous Structural Changes to the Inner Nuclear Membrane in HCMV Infected Cells

**Figure 3:**
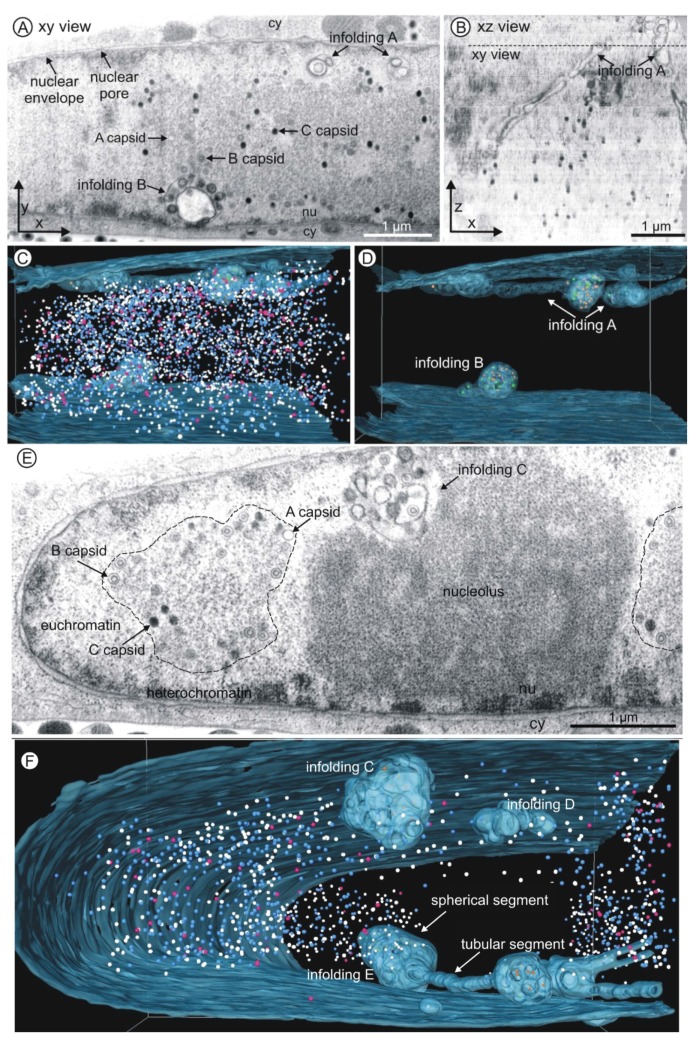
FIB/SEM tomography of an HCMV infected nucleus five days post infection. (**A**–**D**) Volume 1; (**E**–**F**) Volume 2; (**A**,**E**) Single FIB/SEM micrographs. High contrast allows identification of membranes and nuclear capsids. Nuclear membrane structures (infoldings) are visible close to the nuclear envelope (see also Movies S1 and S2). nu nucleoplasm, cy cytoplasm. Dimensions of each volume: 6.4 µm × 5.5 µm × 5 µm; (**B**) Xz view of the tomogram of volume 1. The dashed line marks the position of the FIB/SEM image shown in (**A**). (**C**,**D**,**F**) Three-dimensional reconstructions of the inner nuclear membrane with infoldings (blue, semi-transparent) and capsids (nucleoplasm: A capsids pink; B capsids blue; C capsids white; infoldings: A capsids purple; B capsids orange; C capsids green). See also Movies S3 and S4. (C and D) Volume 1 contains 4160 capsids. (**E**) The dashed line marks a replication center. (**F**) Infolding D has no connection to the inner nuclear membrane. Infolding E consists of tubular and spherical segments. Volume 2 contains 1338 capsids.

**Figure 4:**
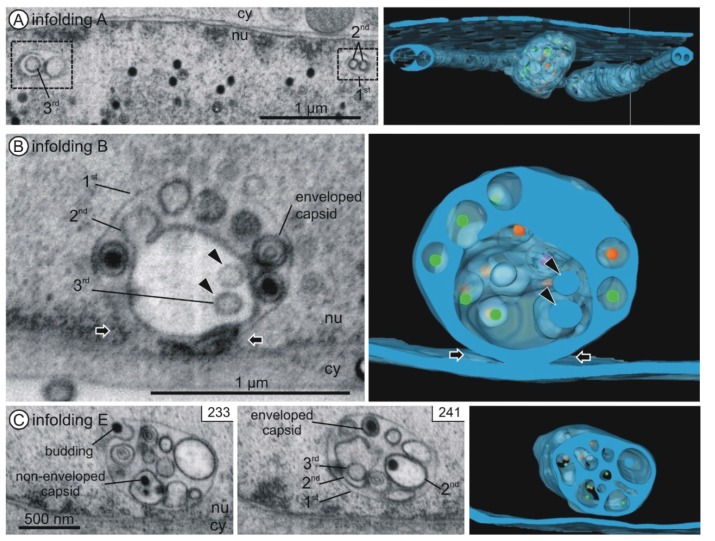
Internal structure of nuclear infoldings. Left and middle panels: FIB/SEM micrographs of the reconstructed details. See also Movies S1 and S2. Right panels: Three-dimensional reconstructions. Nuclear infoldings (semi-transparent blue) and capsids inside the infoldings (A capsids purple; B capsids orange; C capsids green). (**A**) Infolding A. Cross sections through two tubular segments (1st order infoldings) with 2nd order infoldings and a 3rd order infolding. Three-dimensional imaging reveals the tubular shape of the 2nd order infoldings and the spherical shape of the 3rd order infolding; (**B**) Connection of infolding B with the inner nuclear membrane (black arrows). Vesicles (2nd order infoldings) and three enveloped capsids are visible within the 1st order infolding. The 2nd order infolding contains two 3rd order infoldings (black arrowheads); (**C**) Infolding E with 1st, 2nd and 3rd order infoldings, a budding capsid, enveloped capsids in 1st order infoldings and non-enveloped capsids in 2nd order infoldings. nu nucleoplasm, cy cytoplasm.

**Figure 5:**
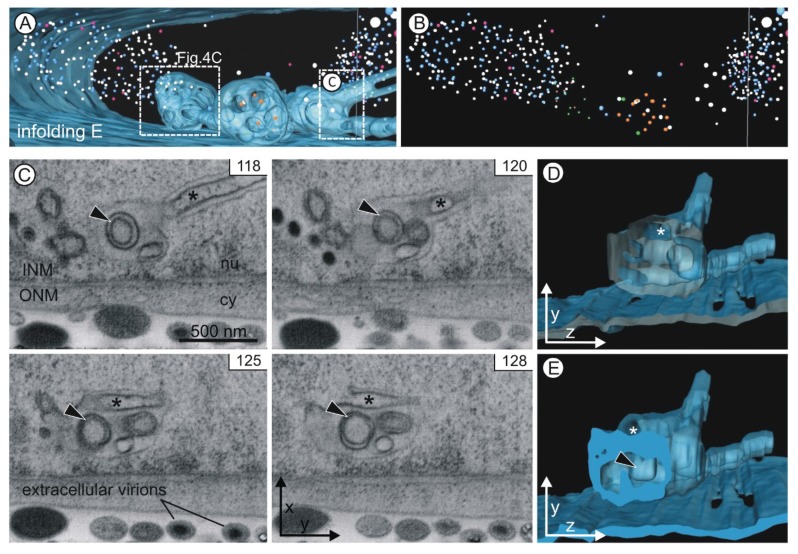
Infolding E. (**A**) Two large spherical segments (diameters 1 µm) are connected by a tubular segment (diameter ~180 nm). A smaller spherical segment at the front branches out into two tubular segments. The boxes mark the position of the details depicted in Figure 4C and Figure 5C–E; (**B**) Presentation of the capsids alone shows the cloud-like structure of the nucleoplasmic capsids; (**C**) A 3rd order infolding (arrowhead) is visible in a sequence of more than ten images (see Movie S4). The images also show an invagination within the tubular segment (asterisk). Its connection with the nucleoplasm is evident in image 128. INM inner nuclear membrane, ONM outer nuclear membrane, nu nucleoplasm, cy cytoplasm; (**D**,**E**) The yz view of the three-dimensional model shows the opening of the invagination (asterisk). The model is depicted without capsids.

With the three-dimensional imaging approach we could reveal an unpredicted high complexity of the intranuclear membrane structures in HCMV-infected cells with respect to their external appearance (Figure 3) and internal structure (Figure 4 and Figure 5).

The representative two-dimensional images of volume 1 (Figure 3A) and volume 2 (Figure 3E) showed membrane structures within the nucleus of the HCMV infected fibroblast. One of the membrane structures (infolding B, in more detail in Figure 4B) clearly showed a connection with the nuclear envelope proving that it was formed by infolding of the inner nuclear membrane. It is reasonable to assume that all visible intranuclear membranes resulted from nuclear infoldings as it has been previously described [1]. One two-dimensional image of volume 2 (Figure 3E) showed one nuclear membrane structure (1st order infolding) filled with vesicles (2nd order infoldings) and capsids (Figure 3E, infolding C).

The high resolution in the z direction (20 nm, Figure 3B) allowed detailed reconstruction of intranuclear membrane structures and nuclear HCMV capsids in three dimensions (Figure 3C,D,F). This revealed that all nuclear infoldings were located close to the nuclear envelope. Infolding D (Figure 3F) showed no connection to the inner nuclear membrane. Some infoldings protruded outside of the visualized volumes (Figure 3D, infolding A; Figure 3F, infolding E).

First order infoldings could be described as either spherical (diameter up to 1 µm) or tubular (length up to several micrometers and a diameter of ~200 nm). Some infoldings consisted of one single spherical segment (Figure 3F, infolding C), whereas other spherical segments were interconnected by tubular segments (Figure 3D, infolding A; Figure 3F, infolding E). Furthermore, segments could also branch (Figure 3F, infolding E). The tomograms and the three-dimensional reconstructions can be found in the Supplemental Materials (Movies S1–S4).

### 3.3. 1st, 2nd and 3rd Order Infoldings

All nuclear infoldings in the two-dimensional micrographs contained various vesicles and most of them also contained capsids (Figure 3, Figure 4 and Figure 5). Only after detailed analysis by three-dimensional FIB/SEM imaging we discovered a hierarchical organization of the nuclear infoldings (Figure 4 and Figure 5): All 1st order infoldings contained at least 2nd order infoldings. Some 2nd order infoldings could clearly be identified as originating from invaginations into 1st order infoldings (Figure 5C, image 128). Within the 2nd order infoldings again 3rd order infoldings could be identified (Figure 4 and Figure 5C, black arrowheads). Sometimes 2nd order infoldings also contained viral capsids (Figure 4C).

The lumen of 1st order infoldings equals the perinuclear space. The lumen of 2nd order infoldings originated from invaginations of nucleoplasm into 1st order infoldings, which is in line with the finding that the membrane of 2nd order infoldings was never continuous with the outer nuclear membrane. The lumen of 2nd order infoldings was also often less electron dense than the perinuclear space (Figure 4) and never showed a cytoplasmic texture. The lumen of 3rd order infoldings again had a similar electron density as the perinuclear space (Figure 4B).

**Figure 6:**
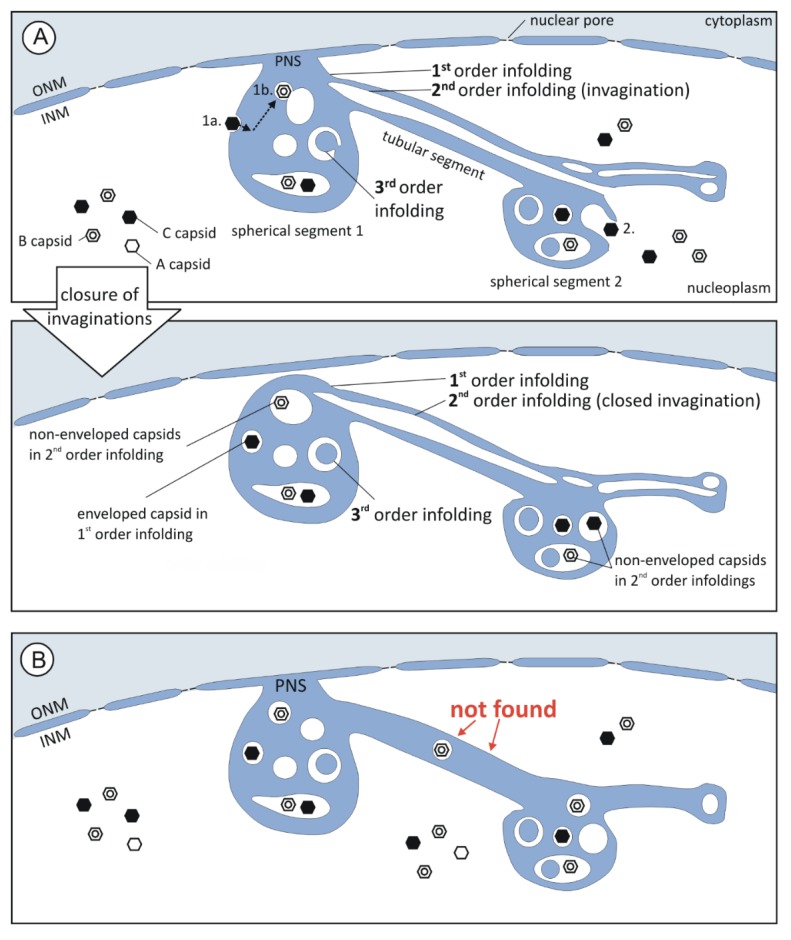
Summary of the architecture of intranuclear membrane structures revealed by FIB/SEM tomography. (**A**) Model describing infolding formation: The hierarchy of the compartments (1st, 2nd, and 3rd order infolding) could be explained by invagination of membrane into the respective compartment and subsequent membrane fission. Thus, the lumen of 1st and 3rd order infoldings equals the perinuclear space (PNS, blue) and the lumen of 2nd order infoldings the nucleoplasm (white). Non-enveloped capsids were only found in the nucleoplasm and 2nd order infoldings, enveloped capsids only in the lumen of 1st order infoldings; (1a.) It seemed obvious that enveloped capsids result from capsid budding into 1st order infoldings. (1b.) Capsid envelopes might be able to fuse with membranes of 2nd order infoldings, resulting in non-enveloped capsids in 2nd order infoldings; (2.) Alternatively, non-enveloped capsids in 2nd order infoldings could result from capsid trapping during formation of invaginations; (**B**) We never found tubular segments with capsids. Tubular segments always contained 2nd order infoldings.

Tubular segments of 1st order infoldings always contained one or more tubular 2nd order infoldings (Figures 4A and 5C), *i.e.*, invaginations, which always partially overlapped with each other (Movies S1 and S2). In five cases, the invaginations were found to be open at one end (Figure 5E and Figure 6A top) and in six cases they were closed at both ends, showing no connection to the nucleoplasm (Figure 6A bottom), but they were never open on both ends (Movies S1 and S2 in supplemental file). Some of the tubular segments contained 3rd order infoldings but never capsids. The general architecture of the infoldings is schematically summarized in Figure 6.

Our three-dimensional FIB/SEM data represent a subvolume of an infected nucleus at five days post infection. Prior to FIB/SEM tomography we performed standard TEM analysis from 13 independent experiments and one serial sectioning TEM experiment with the same infected cell culture as used for FIB/SEM imaging, to ensure that this subvolume allowed a representative three-dimensional analysis.

**Figure 7:**
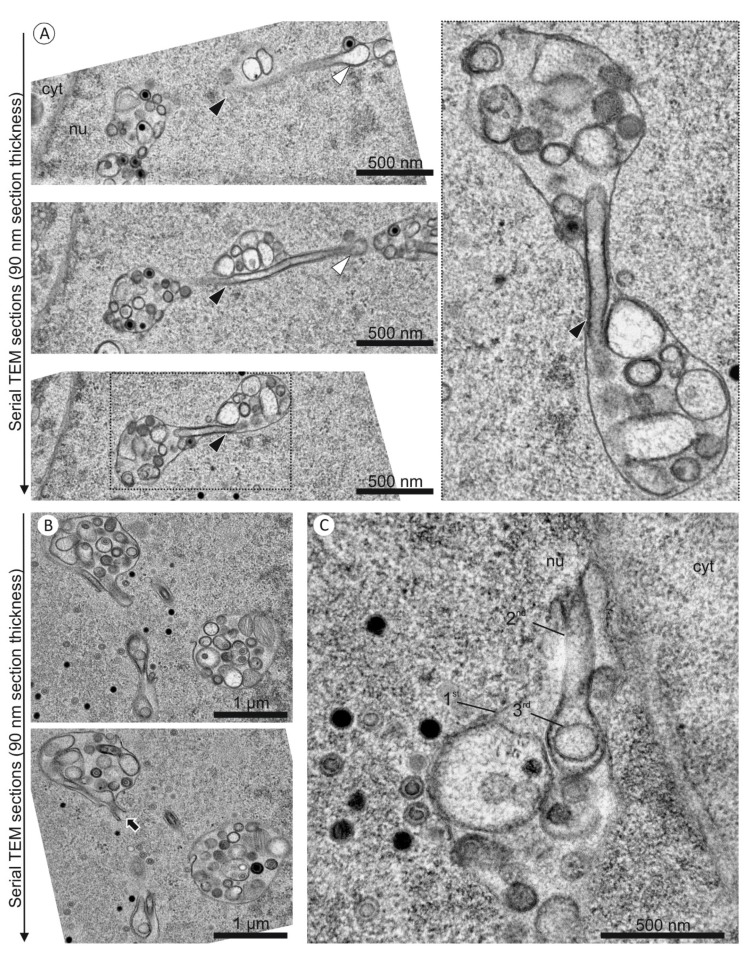
(Serial-)TEM images of nuclear infoldings in HCMV infected fibroblasts at five days post infection. (**A**,**B**) The sample used for FIB/SEM tomography was objected to serial ultrathin sectioning. The images are captured from two different nuclei. (**A**) Three subsequent images through an infolding show a tubular segment with a 2nd order infolding (arrowheads); (**B**) The lower image shows an opening of a 2nd order infolding towards the nucleoplasm (arrow); (**C**) A standard TEM image of an independent experiment also shows the opening of a 2nd order infolding towards the nucleoplasm.

Standard and serial TEM images of more than 40 nuclei consistently showed nuclear infoldings with nested membranes (Figure 7). The high resolution in the z direction by FIB/SEM allowed a completely new insight into these complex intranuclear structures and enabled us to finally interpret two-dimensional TEM images.

**Figure 8:**
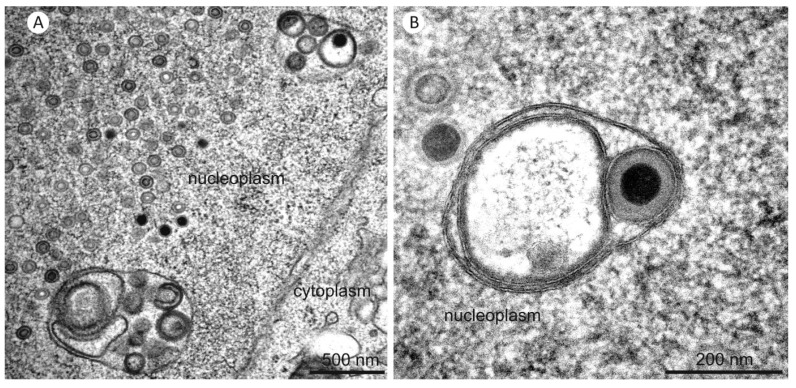
TEM images of nuclear infoldings in two HCMV infected fibroblast nuclei at three days post infection.

TEM imaging was also used to show that nuclear infoldings were already present at three days post infection with no apparent structural difference (Figure 8). At this time nuclear capsids started accumulating in the nucleoplasm and could also be found in infoldings.

### 3.4. Three-Dimensional Distribution of Nuclear HCMV Capsids

Analysis of the quantitative distribution of HCMV capsids in infected nuclei can be performed in detail by FIB/SEM tomography since the resolution in z direction is considerably higher than the diameter of a capsid (about 85 nm). Therefore, all nuclear capsids, outside or inside the infoldings, could be visualized in the three-dimensional reconstructions (Figure 3C,F, Movies S3 and S4). In the nucleoplasm the capsids were primarily located at the periphery of the replication center (Figure 3A,E, dashed line) where viral DNA synthesis takes place [46]. In volume 1, HCMV capsids seemed to be homogeneously distributed throughout the entire volume (Movie S3), whereas in volume 2 it became evident that the nuclear architecture influenced the distribution of the capsids since the nucleolus occupied a big portion of the nucleoplasm (Movie S4). Consequently, volume 1 contained approximately three times more nuclear capsids than volume 2 (Table 1). Quantification of the number of capsids also enabled us to estimate the productivity of one HCMV infected fibroblast. 5498 capsids were counted in the two volumes.

**Table 1:** Quantification of capsids in volume 1 and 2. The total number of capsids in volume 1 was approximately three times higher than in volume 2 but the ratios of the three capsid types were similar. 97.7% of the total capsids were located within the nucleoplasm. The ratios of A, B and C capsids are given for the nucleoplasm and the infoldings. The capsids in the infoldings were again divided into enveloped capsids in 1st order infoldings and non-enveloped capsids in 2nd order infoldings.

	Number of Capsids (% of Total Capsids)		Distribution (%)		
			A Capsids	B Capsids	C Capsids
**Total Capsids**	5498	(100.0%)	6.0	52.0	42.0
-Volume 1	4160	(75.7%)	6.0	52.9	41.1
-Volume 2	1338	(24.3%)	5.9	49.3	44.8
**In Nucleoplasm**					
(Non-Enveloped)	5370	(97.7%)	6.0	51.8	42.2
**In Infoldings**					
- Enveloped in 1st Order Infoldings	45	(0.8%)	0.0	57.8	42.2
- Non-Enveloped in 2nd Order Infoldings	83	(1.5%)	6.0	66.3	27.7

### 3.5. Quantification of Capsids in Infoldings

We further investigated the distribution of the capsids with respect to the infoldings. Capsids were located either non-enveloped in the nucleoplasm, as enveloped capsids in 1st order infoldings, or as non-enveloped capsids in the lumen of 2nd order infoldings (Figure 6A). We assume that capsids in 1st order infoldings were the result of budding at the inner nuclear membrane (Figure 6A(1a.)). We wanted to test whether budding into infoldings was selective for mature DNA-filled C capsids as shown for the alphaherpesvirus pseudorabies virus (PrV) [47]. In FIB/SEM images, A, B, and C capsids could be identified unambiguously by their structural features (Figure 3A,E).

We first evaluated the ratios of A, B and C capsids in the nucleoplasm and in infoldings. In the nucleoplasm we found 6% A capsids, 52% B capsids and 42% DNA-filled C capsids (Table 1). Although the total capsid numbers differed greatly in volume 1 and 2, the ratios were very similar. In 1st order infoldings the ratio of enveloped C capsids was identical to that in the nucleoplasm, whereas we did not detect enveloped A capsids and therefore a slightly higher percentage of B capsids (Table 1). The similarity of the ratios of C capsids in the nucleoplasm and in the 1st order infoldings suggested that budding at the inner nuclear membrane is not specific for DNA-filled C capsids. Overall, we found only 0.8% of the entire capsids enveloped in 1st order infoldings.

Non-enveloped capsids within 2nd order infoldings could result from de-envelopment of enveloped capsids in 1st order infoldings (Figure 6A(1b.)). Alternatively, non-enveloped capsids from the nucleoplasm could get caught during formation of invaginations into 1st order infoldings. After subsequent closure of the invagination the non-enveloped capsids would be located inside a 2nd order infolding (Figure 6A(2.)). To gain data supporting one of the two hypothetical routes, we compared the capsid ratios in the 1st order infoldings with the ratios in the 2nd order infoldings. The major difference between the two compartments was that no enveloped A capsids could be found in 1st order infoldings, whereas non-enveloped A capsids were present in 2nd order infoldings. Furthermore, we found a lower percentage of C capsids in 2nd order infoldings than in 1st order infoldings. This could indicate that entry of capsids from 1st order infoldings into 2nd order infoldings is specific for immature A and B capsids.

Comparison between nucleoplasm and 2nd order infoldings showed a lower percentage of C capsids and a higher percentage of B capsids in 2nd order infoldings which argues against random trapping of capsids from the nucleoplasm.

Our quantification did not allow a conclusive statement about the route of capsids from the nucleoplasm through the infoldings of different orders into the cytoplasm. However, we could state that entry into 1st order infoldings is in line with the envelopment/de-envelopment model for HCMV nuclear egress and that this route towards the cytoplasm did not seem to be specific for DNA-filled C capsids.

## 4. Discussion

### 4.1. Three-Dimensional Imaging Reveals a Hierarchical Infolding Structure

Our study confirms that the nuclear organization is drastically changed upon HCMV infection. In previous studies, nuclear membrane structures which were induced by herpesviruses or herpesvirus proteins were described [1,48,49,50], but their three-dimensional structure remained hypothetical as well as the reason for their formation and their potential function remained enigmatic. FIB/SEM tomography proved to be an ideal method for detailed three-dimensional analysis of nuclear membrane structures since it provides high resolution in all three dimensions and large volumes in the range of several micrometers can be analyzed quantitatively. By using FIB/SEM tomography, we could show for the first time that the intranuclear membrane structures of an HCMV infected fibroblast are much more complex than expected based on two-dimensional studies [1]. The structural data allows us to develop hypotheses about infolding formation and function. In particular, the analysis of their internal structure with respect to the structural hierarchy, namely 1st, 2nd, and 3rd order infoldings, led to a completely new insight into the formation of the infoldings. From several TEM studies and a serial-sectioning experiment (Figure 7), we can confirm that the nucleus in our FIB/SEM study is representative for HCMV infected fibroblast nuclei.

HCMV induced local disruption of the nuclear lamina [23,24,25] creates areas in which the inner nuclear membrane is no longer stabilized by the nuclear lamina. We propose that the lamin disruption could not only provide individual budding sites for nuclear capsids at the inner nuclear membrane but also facilitate the formation of 1st order infoldings [1]. This is in line with earlier observations that changes in nuclear lamin, e.g., in laminopathies, lead to dramatic alterations in the structure of the nuclear membrane and to formation of NR [29,51]. Second order infoldings with open contact to the nucleoplasm (Figure 5C–F) indicated that they are most probably formed by invaginations into 1st order infoldings (Figure 6A). This is also supported by the total absence of 2nd order infoldings that were open on both sides and the similar electron densities of the nucleoplasm and the lumen of 2nd order infoldings. We suggest that occasional fusion and fission of 2nd order membranes lead to closed 2nd order invaginations (Figure 6A). 3rd order infoldings (Figure 3, Figure 4 and Figure 5) presumably originate from invaginations of perinuclear space into 2nd order infoldings. The matching of electron densities of the lumen of 1st and 3rd order infoldings can support this assumption (Figure 4). Roffman *et al.* [52] described structures similar to the 2nd order infoldings and interpreted them as “tegusomes”. However, the “tegusomes” contained cytosolic markers such as ribosomes, implying that the infoldings are formed by the inner and outer nuclear membrane. According to the nomenclature of Malhas *et al.* they would be classified as NR type II [29]. In our study, however, the infoldings were only formed by the inner nuclear membrane and we never observed a texture in the 2nd order infolding lumen resembling the cytoplasm (containing ribosomes or cytoskeletal elements).

Taken together, we could clearly show that the HCMV induced membrane structures are extremely complex type I NR. Additionally to being “branched” or “extremely ramified” [29] the novelty of the here described structure is its extreme tendency to invaginate into itself, causing an hierarchical architecture. This renders the infoldings an unprecedented intranuclear membrane structure.

### 4.2. The Pushing Membrane Model

Concerning the driving force for formation of such complex nuclear infoldings we suggest the *pushing membrane model*: As shown for HSV-1 morphogenesis in Vero cells, a large quantity of membranes is required for maintaining membrane integrity of the enlarging nucleus and cell as well as to provide a sufficient amount of phospholipids for viral envelopment [53]. The authors of the study examined *de novo* synthesis of phospholipids in the course of HSV-1 infection and found that membrane synthesis does not stop after nuclear enlargement. We hypothesize that this is also the case in HCMV infection. Excess of newly synthesized membrane might then be pushed from the endoplasmic reticulum via nuclear pores towards the inner nuclear membrane. Due to local lamin disruptions the inner nuclear membrane might then be pushed into the nucleoplasm, forming a 1st order infolding (“*pushing membrane model*”). At this point the infolding might then either increase in size or invaginate into its own lumen to form a 2nd order infolding. Since we found no infolding located along the more convex lateral margins of the nuclear envelope (Figure 3F), we suggest that curved membranes might be more resistant against infolding formation since the lipid molecules are probably better stabilized by the surrounding lipid molecules, similar to the stability of masonry arches.

Formation of 2nd order infoldings could be induced non-specifically by obstacles (e.g., replication center or nucleoli) which limit the space for expansion of 1st order infoldings. This is supported by the restriction of the infoldings to the periphery of the nucleoplasm. Alternatively, an undertow that builds up inside the 1st order infolding caused by the constant movement of the infolding membrane away from the inner nuclear membrane can pull the apex of the infolding into the infolding lumen. We also found indications that invagination into 1st order infoldings could be initiated specifically by proteins that mediate membrane curvature during primary envelopment of nuclear capsids. In TEM images as well as in some FIB/SEM images, we frequently observed a higher electron density and thickening of (invaginating) membranes of 2nd order infoldings, suggesting the presence of proteins at these sites (Figure 4C, image 233 and Figure 8). It was shown that rabbit kidney cells which co-express the PrV proteins pUL31 and pUL34 (homologs to HCMV pUL50 and pUL53) form vesicles within the perinuclear space (=2nd order infoldings) in the absence of infection [48,49]. These vesicles exhibited a structure very similar to the vesicular membrane structures we observed.

The high complexity of the HCMV-induced infoldings of the inner nuclear membrane indicates that infolding formation is an abundant and highly dynamic process which seems to be halted only when the space inside an infolding is fully occupied.

### 4.3. Three-Dimensional Analysis of Nuclear HCMV Capsids

Our three-dimensional approach furthermore enabled us to analyze the distribution and number of nuclear capsids in great detail. Wild *et al.* [54] estimated that in MDBK cells “…more than 1000 (herpesvirus) capsids may be produced within a single nucleus”. We found 5498 capsids only in a part of the HCMV infected nucleus, significantly more than expected. On the basis of the above figures, we estimate that roughly 15,000–30,000 nuclear capsids were present in the entire nucleus at the moment of high-pressure freezing.

Concerning the amounts and distribution of A, B and C capsids in the nucleoplasm, our data is in line with previous two-dimensional studies [12,55] in which the majority of nuclear capsids were B capsids and all three capsid types were favorably located at the replication center. Considering the high number of capsids that reach the cytoplasm, the number of enveloped capsids in 1st order infoldings, which amounts to less than 1% of the total number of nuclear capsids, was unexpectedly low.

Our data about capsids within infoldings are in line with the envelopment/de-envelopment model for nuclear egress of HCMV. Enveloped capsids in 1st order infoldings result from budding of nucleoplasmic capsids at the inner nuclear membrane (Figure 4C and Figure 6A(1a.)) and these capsids are able to reach the cytoplasm through de-envelopment at the outer nuclear membrane. We could not detect a selectivity for budding of DNA-filled C capsids into the infoldings. This is in contrast to the predominance of C capsids in the perinuclear space described for PrV [47], but our result is in line with studies on other herpesviruses that report non-specific nuclear egress (reviewed in [19]). The predominance of cytoplasmic [4,5,12,41] and extracellular (Figure 5C, bottom left; Movies S1 and S2) C capsids, however, suggests the existence of a sorting mechanism that would thus apply after capsid budding at the inner nuclear membrane.

The origin of capsids in 2nd order infoldings is not as clear. Two different routes may be possible: (I) de-envelopment of enveloped capsids on 2nd order infolding membranes (Figure 6A(1b.)), and (II) accidental trapping of non-enveloped nucleoplasmic capsids during formation of invaginations into 1st order infoldings (Figure 6A(2.)). Independent of the route on which capsids reach 2nd order infoldings, higher ratios of non-enveloped A and B capsids in 2nd order infoldings compared to the nucleoplasm and 1st order infoldings could suggest a selectivity for immature A and B capsids to enter 2nd order infoldings. Assuming that capsids in 2nd order infoldings cannot reach the cytoplasm we could hypothesize that this might be part of a sorting mechanism leading to a higher abundance of C capsids in the cytoplasm. This could provide an interesting working hypothesis for further experiments.

### 4.4. Function of the Nuclear Infoldings

The hypothesis about nuclear infoldings as part of the nuclear egress route for HCMV capsids is originally based on the finding that the infoldings enlarge the inner nuclear membrane area and appear to be free of nuclear lamina [1]. This would provide a large area for budding of nuclear capsids into the perinuclear space. With our new data we confirm the enlargement of the inner nuclear membrane; however, the absence of the lamin layer remains to be formally proven.

We revealed that all infoldings were located at the periphery of the nucleoplasm (Figure 3D,F) but still in close proximity to the accumulations of nuclear capsids around the replication center. This supports the earlier assumption that the infoldings might serve as egress route for capsids from the nucleus [1]. The infoldings can also reach deep into the nucleoplasm as shown by an X-ray tomographic study of epithelial-like kidney cells stably co-transfected with the PrV proteins pUL31 and pUL34-GFP. The pUL34 positive fluorescent “speckles” representing perinuclear vesicles were not only located near the nuclear envelope [48]. On the other hand, we never detected enveloped capsids within the tubular segments of 1st order infoldings (Movies S1 and S2, Figure 6B). Instead, the tubular segments were always completely occupied by higher order infoldings (Figure 6A) which could be considered as obstructions hindering the movement of capsids towards the outer nuclear membrane. Additionally, we described infoldings without a connection to the inner nuclear membrane (Figure 3F, infolding D). The capsids in these infoldings have no access to the outer nuclear membrane and, consequently, no access to the cytoplasm.

## 5. Conclusions

From these (static) structural observations, it is reasonable to conclude that the nuclear membrane structures do not necessarily play a major role for nuclear egress of HCMV capsids. In this case, their formation might be explained by a side effect of HCMV infection associated with massively enhanced phospholipid synthesis (“*pushing membrane model*”). However, the intranuclear membrane structures might be highly dynamic so that they allow constant movement of capsids and vesicles along the infoldings from the nucleoplasm towards the outer nuclear membrane. Our study provides many starting points for further experiments. For further studies it is especially important to examine the infoldings and nuclear egress in a dynamic manner in order to answer the question of their formation and their function.

Our data confirm that capsid budding into the perinuclear space occurs on the large infoldings [1]. The structural analysis using FIB/SEM tomography additionally provided us with exciting insights into nuclear infoldings that make it possible to hypothesize about the mechanism of their formation. Furthermore, the high number of HCMV capsids gave us a new idea about the enormous productivity of an HCMV-infected fibroblast.

## Supplementary Materials

Movie S1 and S2 (related to Figure 3 and Figure 4). FIB/SEM tomograms of the two volumes of an HCMV infected nucleus. The respective FIB/SEM image stacks consist of 250 individual images each. The dimensions of each dataset are 6.4 µm × 5.5 µm × 5 µm. S1: Volume 1 (Figure 3A–D). S2: Volume 2 (Figure 3E,F). Movie S3 and S4 (related to Figure 3). Three-dimensional reconstructions of the two volumes of an HCMV infected nucleus. The reconstructions of the nuclear membrane are shown with the intranuclear membrane structures (semi-transparent blue), capsids in the nucleoplasm (A capsids pink; B capsids blue; C capsids white) and capsids within infoldings (A capsids purple; B capsids orange; C capsids green). S3: Volume 1 (Figure 3A–D). Note the homogeneous capsid distribution throughout the entire nucleoplasm. S4: Volume 2 (Figure 3E,F). Note the cloud-like capsid distribution.

## References

[1] Buser C., Walther P., Mertens T., Michel D. (2007). Cytomegalovirus Primary Envelopment Occurs at Large Infoldings of the Inner Nuclear Membrane. J. Virol..

[2] Homman-Loudiyi M., Hultenby K., Britt W., Soderberg-Naucler C. (2003). Envelopment of Human Cytomegalovirus Occurs by Budding into Golgi-Derived Vacuole Compartments Positive for gB, Rab 3, Trans-Golgi Network 46, and Mannosidase II. J. Virol..

[3] Jiang X.J., Adler B., Sampaio K.L., Digel M., Jahn G., Ettischer N., Stierhof Y.-D., Scrivano L., Koszinowski U., Mach M. (2008). UL74 of human cytomegalovirus contributes to virus release by promoting secondary envelopment of virions. J. Virol..

[4] Meissner C.S., Suffner S., Schauflinger M., von Einem J., Bogner E. (2011). A Leucine Zipper Motif of a Tegument Protein Triggers Final Envelopment of Human Cytomegalovirus. J. Virol..

[5] Schauflinger M., Fischer D., Schreiber A., Chevillotte M., Walther P., Mertens T., von Einem J. (2011). The Tegument Protein UL71 of Human Cytomegalovirus Is Involved in Late Envelopment and Affects Multivesicular Bodies. J. Virol..

[6] Seo J.-Y., Britt W.J. (2007). Cytoplasmic Envelopment of Human Cytomegalovirus Requires the Postlocalization Function of Tegument Protein pp28 within the Assembly Compartment. J. Virol..

[7] Wang J., Loveland A.N., Kattenhorn L.M., Ploegh H.L., Gibson W. (2006). High-Molecular-Weight Protein (pUL48) of Human Cytomegalovirus Is a Competent Deubiquitinating Protease: Mutant Viruses Altered in Its Active-Site Cysteine or Histidine Are Viable. J. Virol..

[8] Gibson W. (1996). Structure and assembly of the virion. Intervirology.

[9] Gibson W. (2008). Structure and formation of the cytomegalovirus virion. Curr. Top. Microbiol. Immunol..

[10] Varnum S.M., Streblow D.N., Monroe M.E., Smith P., Auberry K.J., Pasa-Tolic L., Wang D., Camp D.G., Rodland K., Wiley S. (2004). Identification of Proteins in Human Cytomegalovirus (HCMV) Particles: The HCMV Proteome. J. Virol..

[11] Schmolke S., Kern H.F., Drescher P., Jahn G., Plachter B. (1995). The dominant phosphoprotein pp65 (UL83) of human cytomegalovirus is dispensable for growth in cell culture. J. Virol..

[12] Tandon R., Mocarski E.S., Conway J.F. (2015). The A, B, Cs of Herpesvirus Capsids. Viruses.

[13] Toropova K., Huffman J.B., Homa F.L., Conway J.F. (2011). The herpes simplex virus 1 UL17 protein is the second constituent of the capsid vertex-specific component required for DNA packaging and retention. J. Virol..

[14] Yu X., Trang P., Shah S., Atanasov I., Kim Y.-H., Bai Y., Zhou Z.H., Liu F. (2005). Dissecting human cytomegalovirus gene function and capsid maturation by ribozyme targeting and electron cryomicroscopy. Proc. Natl. Acad. Sci. USA.

[15] Panté N., Kann M. (2002). Nuclear Pore Complex Is Able to Transport Macromolecules with Diameters of ∼39 nm. Mol. Biol. Cell.

[16] Leuzinger H., Ziegler U., Schraner E.M., Fraefel C., Glauser D.L., Heid I., Ackermann M., Mueller M., Wild P. (2005). Herpes Simplex Virus 1 Envelopment Follows Two Diverse Pathways. J. Virol..

[17] Skepper J.N., Whiteley A., Browne H., Minson A. (2001). Herpes Simplex Virus Nucleocapsids Mature to Progeny Virions by an Envelopment → Deenvelopment → Reenvelopment Pathway. J. Virol..

[18] Stackpole C.W. (1969). Herpes-Type Virus of the Frog Renal Adenocarcinoma I. Virus Development in Tumor Transplants Maintained at Low Temperature. J. Virol..

[19] Mettenleiter T.C., Müller F., Granzow H., Klupp B.G. (2013). The way out: What we know and do not know about herpesvirus nuclear egress. Cell. Microbiol..

[20] Schulz K.S., Klupp B.G., Granzow H., Paßvogel L., Mettenleiter T.C. (2015). Herpesvirus nuclear egress: Pseudorabies Virus can simultaneously induce nuclear envelope breakdown and exit the nucleus via the envelopment–deenvelopment-pathway. Virus Res..

[21] Dunn W., Chou C., Li H., Hai R., Patterson D., Stolc V., Zhu H., Liu F. (2003). Functional profiling of a human cytomegalovirus genome. Proc. Natl. Acad. Sci..

[22] Yu D., Silva M.C., Shenk T. (2003). Functional map of human cytomegalovirus AD169 defined by global mutational analysis. Proc. Natl. Acad. Sci..

[23] Marschall M., Marzi A., aus dem Siepen P., Jochmann R., Kalmer M., Auerochs S., Lischka P., Leis M., Stamminger T. (2005). Cellular p32 Recruits Cytomegalovirus Kinase pUL97 to Redistribute the Nuclear Lamina. J. Biol. Chem..

[24] Muranyi W., Haas J., Wagner M., Krohne G., Koszinowski U.H. (2002). Cytomegalovirus Recruitment of Cellular Kinases to Dissolve the Nuclear Lamina. Science.

[25] Sharma M., Kamil J.P., Coughlin M., Reim N.I., Coen D.M. (2014). Human Cytomegalovirus UL50 and UL53 Recruit Viral Protein Kinase UL97, not Protein Kinase C, for Disruption of Nuclear Lamina and Nuclear Egress in Infected Cells. J. Virol..

[26] Hatch E.M., Hetzer M.W. (2012). RNP Export by Nuclear Envelope Budding. Cell.

[27] Speese S.D., Ashley J., Jokhi V., Nunnari J., Barria R., Li Y., Ataman B., Koon A., Chang Y.-T., Li Q. (2012). Nuclear envelope budding enables large ribonucleoprotein particle export during synaptic Wnt signaling. Cell.

[28] Bayer C., Varani S., Wang L., Walther P., Zhou S., Straschewski S., Bachem M., Söderberg-Naucler C., Mertens T., Frascaroli G. (2013). Human Cytomegalovirus Infection of M1 and M2 Macrophages Triggers Inflammation and Autologous T-Cell Proliferation. J. Virol..

[29] Malhas A., Goulbourne C., Vaux D.J. (2011). The nucleoplasmic reticulum: form and function. Trends Cell Biol..

[30] Ballerini M., Milani M., Costato M., Squadrini F., Turcu I.C. (1997). Life science applications of focused ion beams (FIB). Eur. J. Histochem. EJH.

[31] Becker C., Ali K., Knott G., Fua P. (2013). Learning context cues for synapse segmentation. IEEE Trans. Med. Imaging.

[32] Hekking L.H.P., Lebbink M.N., de Winter D.A.M., Schneijdenberg C.T.W.M., Brand C.M., Humbel B.M., Verkleij A.J., Post J.A. (2009). Focused ion beam-scanning electron microscope: Exploring large volumes of atherosclerotic tissue. J. Microsc..

[33] Knott G., Rosset S., Cantoni M. (2011). Focussed ion beam milling and scanning electron microscopy of brain tissue. J. Vis. Exp. JoVE.

[34] Leser V., Drobne D., Pipan Z., Milani M., Tatti F. (2009). Comparison of different preparation methods of biological samples for FIB milling and SEM investigation. J. Microsc..

[35] Schertel A., Snaidero N., Han H.-M., Ruhwedel T., Laue M., Grabenbauer M., Möbius W. (2013). Cryo FIB-SEM: Volume imaging of cellular ultrastructure in native frozen specimens. J. Struct. Biol..

[36] Schroeder-Reiter E., Pérez-Willard F., Zeile U., Wanner G. (2009). Focused ion beam (FIB) combined with high resolution scanning electron microscopy: a promising tool for 3D analysis of chromosome architecture. J. Struct. Biol..

[37] Kizilyaprak C., Daraspe J., Humbel B. (2014). Focused ion beam scanning electron microscopy in biology. J. Microsc..

[38] Villinger C., Gregorius H., Kranz C., Höhn K., Münzberg C., Wichert G., Mizaikoff B., Wanner G., Walther P. (2012). FIB/SEM tomography with TEM-like resolution for 3D imaging of high-pressure frozen cells. Histochem. Cell Biol..

[39] Villinger C., Schauflinger M., Gregorius H., Kranz C., Höhn K., Nafeey S., Walther P. (2014). Three-dimensional imaging of adherent cells using FIB/SEM and STEM. Methods Mol. Biol. Clifton NJ.

[40] Sinzger C., Hahn G., Digel M., Katona R., Sampaio K.L., Messerle M., Hengel H., Koszinowski U., Brune W., Adler B. (2008). Cloning and sequencing of a highly productive, endotheliotropic virus strain derived from human cytomegalovirus TB40/E. J. Gen. Virol..

[41] Schauflinger M., Villinger C., Mertens T., Walther P., von Einem J. (2013). Analysis of human cytomegalovirus secondary envelopment by advanced electron microscopy: HCMV morphogenesis. Cell. Microbiol..

[42] Walther P., Ziegler A. (2002). Freeze substitution of high-pressure frozen samples: the visibility of biological membranes is improved when the substitution medium contains water. J. Microsc..

[43] ImageJ. http://rsbweb.nih.gov/ij/index.html.

[44] Kremer J.R., Mastronarde D.N., McIntosh J.R. (1996). Computer visualization of three-dimensional image data using IMOD. J. Struct. Biol..

[45] The IMOD Home Page. http://bio3d.colorado.edu/imod/.

[46] Strang B.L. (2015). Viral and cellular subnuclear structures in human cytomegalovirus-infected cells. J. Gen. Virol..

[47] Klupp B.G., Granzow H., Mettenleiter T.C. (2011). Nuclear Envelope Breakdown Can Substitute for Primary Envelopment-Mediated Nuclear Egress of Herpesviruses. J. Virol..

[48] Hagen C., Guttmann P., Klupp B., Werner S., Rehbein S., Mettenleiter T.C., Schneider G., Grunewald K. (2012). Correlative VIS-fluorescence and soft X-ray cryo-microscopy/tomography of adherent cells. J. Struct. Biol..

[49] Klupp B.G., Granzow H., Fuchs W., Keil G.M., Finke S., Mettenleiter T.C. (2007). Vesicle formation from the nuclear membrane is induced by coexpression of two conserved herpesvirus proteins. Proc. Natl. Acad. Sci. USA.

[50] Pignatelli S., Monte P.D., Landini M.P., Severi B., Nassiri R., Gilloteaux J., Papadimitriou J.M., Shellam G.R. (2007). Cytomegalovirus Primary Envelopment at Large Nuclear Membrane Infoldings: What’s New?. J. Virol..

[51] Prüfert K., Vogel A., Krohne G. (2004). The lamin CxxM motif promotes nuclear membrane growth. J. Cell Sci..

[52] Roffman E., Albert J.P., Goff J.P., Frenkel N. (1990). Putative site for the acquisition of human herpesvirus 6 virion tegument. J. Virol..

[53] Sutter E., de Oliveira A.P., Tobler K., Schraner E.M., Sonda S., Kaech A., Lucas M.S., Ackermann M., Wild P. (2012). Herpes simplex virus 1 induces *de novo* phospholipid synthesis. Virology.

[54] Wild P., Engels M., Senn C., Tobler K., Ziegler U., Schraner E.M., Loepfe E., Ackermann M., Mueller M., Walther P. (2005). Impairment of Nuclear Pores in Bovine Herpesvirus 1-Infected MDBK Cells. J. Virol..

[55] Tandon R., Mocarski E.S. (2012). Viral and host control of cytomegalovirus maturation. Trends Microbiol..

